# Bacterial Metabolites: A Link between Gut Microbiota and Dermatological Diseases

**DOI:** 10.3390/ijms24043494

**Published:** 2023-02-09

**Authors:** Albert Stec, Mariusz Sikora, Magdalena Maciejewska, Karolina Paralusz-Stec, Milena Michalska, Ewa Sikorska, Lidia Rudnicka

**Affiliations:** 1 Department of Dermatology, Medical University of Warsaw, Koszykowa 82A, 02-008 Warsaw, Poland; 2 National Institute of Geriatrics, Rheumatology and Rehabilitation, Spartańska 1, 02-637 Warsaw, Poland; 3 Department of General, Vascular and Transplant Surgery, Medical University of Warsaw, Banacha 1a, 02-097 Warsaw, Poland; 4 Department of Experimental and Clinical Physiology Center for Preclinical Research, Medical University of Warsaw, Banacha 1b, 02-097 Warsaw, Poland

**Keywords:** microbiota, gut–skin axis, dysbiosis, inflammation, immune-mediated inflammatory diseases, intestinal microbial metabolites, SCFA, TMAO, probiotics, fecal microbiota transplantation

## Abstract

Dysbiosis has been identified in many dermatological conditions (e.g., psoriasis, atopic dermatitis, systemic lupus erythematosus). One of the ways by which the microbiota affect homeostasis is through microbiota-derived molecules (metabolites). There are three main groups of metabolites: short-chain fatty acids (SCFAs), tryptophan metabolites, and amine derivatives including trimethylamine N-oxide (TMAO). Each group has its own uptake and specific receptors through which these metabolites can exert their systemic function. This review provides up-to-date knowledge about the impact that these groups of gut microbiota metabolites may have in dermatological conditions. Special attention is paid to the effect of microbial metabolites on the immune system, including changes in the profile of the immune cells and cytokine disbalance, which are characteristic of several dermatological diseases, especially psoriasis and atopic dermatitis. Targeting the production of microbiota metabolites may serve as a novel therapeutic approach in several immune-mediated dermatological diseases.

## 1. Introduction

The intestinal microbiota are the microorganisms colonizing the gastrointestinal tract, which include more than a thousand distinct species of bacteria, viruses, fungi, and protozoa [1]. The impact of the gut microbiome on human homeostasis has been widely studied, especially its ability to modulate inflammatory responses. The quantitative and qualitative changes in the intestinal microbiota’s composition have been observed in many dermatological conditions, such as psoriasis [2,3,4], systemic lupus erythematosus [5], atopic dermatitis [6,7], and systemic sclerosis [8,9]. This condition is called dysbiosis, and its impact on human health is still not clearly understood. In contrast to the normobiotic state, altered exposure of the host to diverse microbial stimuli may be linked with dysbiosis. Available data suggest that it can be a significantly harmful environmental factor, especially in genetically vulnerable hosts whose endogenous systems, which control inflammation, are already compromised [10].

There are several main mechanisms by which the microbiota could play a role in autoimmunity and stimulation of the immune response: molecular mimicry, an impaired intestinal barrier that may promote translocation of bacterial elements or even whole bacteria, direct interactions with immune cells in Peyer’s patches, and an altered abundance of microbial metabolites with immunomodulatory functions [10,11,12,13]. To date, only some groups of metabolites have been discovered and extensively studied. The most relevant are those that significantly impact the immune system physiology, such as short-chain fatty acids (SCFAs), tryptophan metabolites, and amine derivatives including trimethylamine N-oxide (TMAO) [10,14,15]. These are found to exert particular effects at the cellular and even systemic level by interacting with different receptors in immune and skin cells [16,17,18].

Understanding how microbial metabolites affect the skin and immune cells could be a milestone in deciphering the concept proposed by novel studies regarding the functional link between the intestine and skin, which is called the gut–skin axis [19].

## 2. Short-Chain Fatty Acids

Short-chain fatty acids (SCFAs) are the most studied substances among gut bacterial metabolites. This group consists of aliphatic carboxylic acids with fewer than six carbon atoms. In humans, the three most abundant aliphatic carboxylic acids, which have the highest impact on homeostasis, are acetic, propionic, and butyric acid [16].

SCFAs are produced mostly in the colon due to the fermentation of indigestible dietary fiber mainly by two anaerobic bacterial phyla: *Bacteroides* and *Firmicutes* [20]. Some of the produced SCFAs are absorbed by colonocytes and utilized as an energy source. The rest of the SCFA pool is absorbed into the circulation and exerts systemic effects via four known pathways: interaction with the free fatty acid receptor 2 (FFAR2, also known as GPR43), the free fatty acid receptor 3 (FFAR3, also known as GPR41), the free fatty acid receptor GPR109a, and inhibition of histone deacetylase [16,20].

FFAR2, FFAR3, and GPR109a are G-protein-coupled receptors, and they are expressed in various tissues, including adipocytes, intestinal epithelial cells, pancreatic beta-cells, spleen, and immune cells such as M2 macrophages, neutrophils, eosinophils, and mast cells [16]. As inhibitors of histone deacetylases (HDACs), SCFAs cause an increase in the expression of certain genes in an epigenetic manner. There is a substantial body of evidence confirming that these substances not only act as histone deacetylase inhibitors but can also modify histones directly via so-called histone propionylation and butyrylation [21].

SCFAs’ abilities to modulate the immune system could be beneficial in the prevention, modulation, or prognosis of dermatological disorders resulting from immune system dysregulation. Usami et al. proved that SCFAs, especially butyrate, inhibit the activity of NF-κB in mononuclear cells, which is a transcriptional factor controlling the expression of multiple genes involved in the inflammatory response. Furthermore, it has been shown that SCFAs can markedly reduce the secretion of TNF via the mentioned cells [22]. In macrophages, butyrate reduces the expression of inflammatory cytokines, such as TNF, MCP-1, and IL-6, via HDAC inhibition [23]. In T cells, SCFAs are potent regulators of differentiation and cytokine production. It is known that SCFAs can increase the pool of Treg lymphocytes in an FFAR2- and HDAC-dependent manner [24,25,26]. Additionally, signaling through GPR109a and FFAR2 in dendritic cells (DCs) can lead to the induction of the differentiation of Foxp3^+^ Treg lymphocytes [11,27]. SCFAs also impact B cells. Through HDACs’ inhibitory properties, they can modulate gene expression epigenetically. Specifically, butyrate and propionate decrease the expression of B cells’ *Aicda* and *Prdm1* genes in humans and mice, the products of which are key regulators of antibody class switching and differentiation of B cells [28]. It has been observed that SCFA-fed mice are characterized by decreased IgG1, IgA, and IgE antibody levels, a dampened response to allergens by plasmocytes, and a reduced number of local and systemic class-switched B cells, antibody-forming cells, and class-switched antibodies [28]. The level of SCFAs has also shown a positive correlation with the number of regulatory B cells in rheumatoid arthritis [29]. All the mentioned facts suggest that SCFAs can dampen the immune response and may prevent autoimmune diseases. Valerate, which is a less-studied compound from the SCFA group, also displays tolerogenic properties. It induces IL-10 production in both B and T CD4^+^ cells and suppresses Th17 lymphocytes via a reduction in IL-17 production and downregulation of Th17-associated genes. It also mediates the generation of Breg cells [30,31]. 

In contrast to this result, SCFAs can boost the generation of Th1 and Th17 cells during active immune responses and increase the cytotoxic activity and production of IL-17 via CD8 T cells [23]. These discrepancies in the function of SCFAs can be explained by the host state. In a steady state, SCFAs seem to present tolerogenic properties, but in acute immune responses, they stimulate the response of the immune system to fight the infection more effectively.

### 2.1. Atopic Dermatitis and Hypersensitivity Reactions

There is mounting evidence that SCFAs can interfere with allergy response on many levels. The most crucial function of SCFAs in the pathogenesis of hypersensitivity is their ability to improve intestinal and epithelial barriers. A disrupted epithelial barrier creates favorable conditions for allergen ingress and further sensitization, which is a pivotal feature of atopic dermatitis (AD) [32]. It has been observed in a mouse model of AD that orally administered SCFAs, particularly butyrate, can decrease transepidermal water loss (TEWL) and increase the level of cholesterol and ceramides, particularly ester-linked omega-hydroxy ceramides, in the stratum corneum [33]. The deficiency of these ceramides is linked with AD [34]. What is more, the administration of butyrate was found to affect keratinocytes directly by promoting terminal differentiation. Butyrate-treated human epithelial keratinocytes have displayed increased numbers of “late” differentiation markers such as involucrin, filaggrin, and calmodulin-like skin protein as well as “early” differentiation markers such as desmoglein-1, keratin-1, and keratin-10 [33].

Interestingly, there is ample evidence that AD is characterized by gut dysbiosis, especially the depletion of butyrate-producing bacteria [35,36,37,38]. The reduction in butyrate-producing *Bacteroides fragilis* has been found to correlate with increased total IgE, egg-IgE, and milk-IgE [38]. Moreover, the microbiota in AD produce fewer SCFAs, particularly propionate and butyrate, which have been assessed in fecal samples [38,39]. Reduced exposure to SCFAs early in life may be one of the factors responsible for the development and course of the disease, which seems to confirm the work of Roduit et al. They found that in a one-year-old pediatric population, a lower fecal level of propionate and butyrate was associated with a markedly higher prevalence of atopic diseases diagnosed at the age of 6 [40]. Other studies seem to confirm these results by showing that lower fecal butyrate levels in one-year-old patients are associated with the development of eczema [41,42,43]. Additionally, decreased exposure to valerate is supposedly correlated with a lower prevalence of eczema in children [44,45]. Dysbiosis and decreased SCFA formation might be an effect of prenatal and early-life exposure to antibiotics. As reported by recent large cohort studies, both prenatal and early-life exposures to antibiotics are linked with an increased risk of developing AD [46,47]. A study on a mouse model of AD showed corresponding results. Administration of antibiotics before sensitization was associated with a more severe disease course, increased expression of IL-4, increased levels of IgE, decreased levels of acetate, propionate, and butyrate, and decreased Foxp3^+^ Treg cells [48]. Like in psoriasis, Treg lymphocytes have a significant role in the pathogenesis of atopic diseases [49], which could be at least partially improved by SCFAs.

The tolerogenic effect of SCFAs may be responsible for the acquisition of allergen tolerance. The pathogenesis of human food allergy includes an increased number of intestinal Th2 cells and type 2 innate lymphoid cells (ILC2s), which generate cytokines including IL-4, IL-5, and IL-13 after exposure to food allergens [50]. It is well known that IL-4 actively promotes B cells’ development into plasma cells that synthesize IgE [51]. Cross-linking of allergen-specific IgE on mast cells via FcεRI can elicit degranulation of mast cells after exposure to a particular allergen, which results in an allergic reaction [51]. Many studies report that SCFAs have a direct impact on the majority of cells involved in allergic responses. In mast cells, SCFAs act epigenetically by altering the signaling cascade mediated by FcεRI, which can dampen allergic responses [52,53,54]. These findings are supported by the fact that fecal concentrations of acetate, propionate, and butyrate are lower in patients suffering from IgE-mediated food allergies [55]. Interestingly, like in the case of atopic dermatitis, lower exposure to SCFAs in early childhood might result in the development of food allergy, and also IgE-associated diseases such as asthma [40,42,45]. Moreover, it seems that the protective effect of SCFAs is distinctively observable in IgE-related conditions, contrary to IgE-independent sensitizations [56]. However, sodium butyrate (SB) injected subcutaneously or administered topically suppressed both the elicitation phase and ongoing hypersensitivity reaction in a mouse model sensitized to 2,4,6-trinitro-1-chlorobenzene, which is model for contact dermatitis [57,58]. Sections from areas exposed to SB were characterized by an increased number of Treg lymphocytes, and greater expression of IL-10 and the Foxp3 transcription factor compared to sections from the control tissue [57]. The immunomodulatory effect of SB is histone-acetylation-dependent. Moreover, similar findings were observed in human skin biopsies; after exposure to SB, more Treg cells, increased transcription of Foxp3 and IL-10 genes, and decreased transcription of the IL-6 gene were observed [57]. The oral administration of butyrate or propionate can alleviate symptoms in the mouse model of allergic airway disease [40,52] and food allergy [59]. Additionally, some studies also suggest that exposure to butyrate and propionate may lead to a reduction in IL-4 secretion, which can potentially restore the skin barrier and suppress the allergic response [60].

### 2.2. Psoriasis

Schwarz et al. provide substantial information about the function of SB, which is the counterpart of the butyrate produced by the microbiota on skin immune cells, and its therapeutic potential in skin disorders. They observed that the injection or topical administration of SB can almost completely reverse imiquimod-induced psoriasis-like lesions in the mice. SB was also able to downregulate inflammatory response, which was manifested by decreased expression of IL-17 and enhanced expression of IL-10 and Foxp3. The anti-inflammatory effect was dependent on Treg lymphocytes [61]. The next part of the study included patients with psoriasis. Compared to the controls, the patients had reduced Treg activity, which was partially restored using SB. Moreover, upregulation of the Foxp3 factor and activation of skin Treg cells were observed. Additionally, in the studied skin, SB reduced the expression of IL-6 and IL-17 and upregulated the expression of IL-10 [61]. Similar results were observed by Krejner et al. They found that in skin samples of psoriatic patients, the expression of FFAR2 and GPR109a were decreased, and the administration of SB upregulated these receptors, reduced the expression of IL-6, IL-17, and increased the expression of IL-10 [62]. Interestingly, some of the drugs used in psoriasis management are potent agonists of these receptors; e.g., dimethyl fumarate exhibits a high affinity to the GPR109a receptor [63,64]. Cyclosporine, another antipsoriatic drug, may increase the intestinal uptake of certain SCFAs, especially butyrate [65].

### 2.3. Connective Tissue Diseases

SCFAs may impact lupus pathogenesis. In systemic lupus erythematosus (SLE) patients, gut dysbiosis, along with a decrease in butyrate-producing bacteria and altered SCFA concentrations, can be observed [66,67]. Oral administration of SCFAs, especially butyrate, in both lupus-prone mouse models MRL/Faslpr/lpr and NZB/W F1 can alleviate symptoms of the disease and can reduce dysbiosis by increasing microbiota diversity [28,68]. In comparison to the control group, the mice that were administered butyrate and propionate orally did not demonstrate IgG1/IgG2a kidney deposition, glomerular damage, or skin lesions [28]. Furthermore, this intervention decreased the number of autoantibodies to dsDNA, RNP/Sm, anti-RNA, histones, and nuclei, and reduced the number of plasmacytes [28].

Systemic sclerosis (SSc) is also characterized by dysbiosis, particularly in butyrate-producing genera, but to date, there are no data on human subjects regarding the influence of SCFAs on SSc [69,70]. However, promising results have been obtained in mice. In the bleomycin-induced systemic sclerosis model, the administration of sodium butyrate causes reduced collagen deposition and α-SMA expression in the skin [71]. Additionally, SB has shown efficacy in reducing lung fibrosis and gut dysbiosis. The in vitro part of this study reported that SB can dampen the profibrotic response induced by TGF-β1 in human dermal fibroblasts (HDFs) [71].

## 3. Tryptophan Metabolites

**Figure 1:**
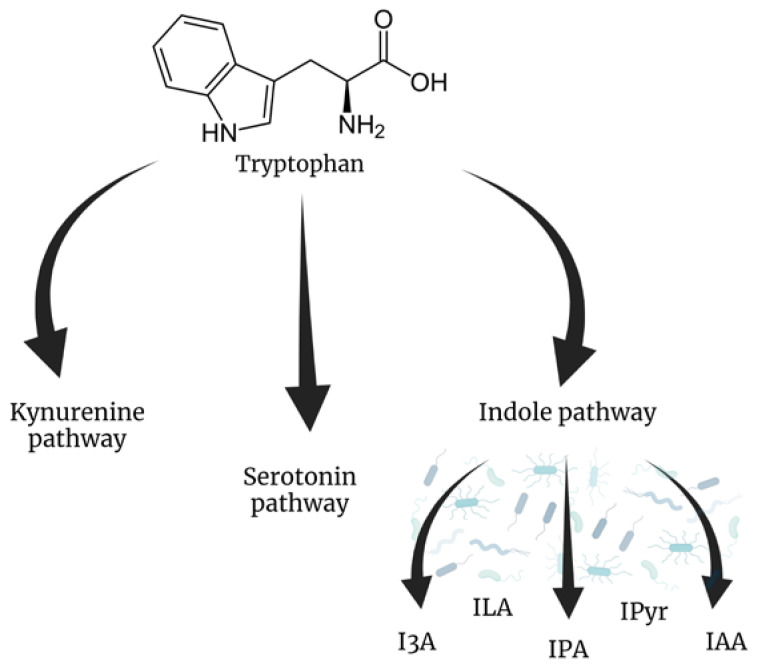
Three pathways of tryptophan metabolism. The indole pathway is mostly associated with intestinal microbiota metabolism. Various compounds produced in this pathway are considered beneficial to human health. I3A—indole-3-aldehyde, ILA—indole lactic acid, IPA—indole propionic acid, IPyr—indole pyruvate, IAA—indole acetic acid. Created with BioRender.com.

In the gastrointestinal tract, dietary proteins are broken down into amino acids. Among them, tryptophan has ample evidence of being utilized by intestinal microbiota. Tryptophan can be metabolized in three general pathways: the kynurenine, serotonin, and indole pathways (Figure 1). The latter is known to be closely associated with microbiota metabolism [72]. Many genera of intestinal bacteria, such as *Clostridium*, *Bacteroides*, *Bifidobacterium*, and *Lactobacillus*, are known to produce tryptophan metabolites [73]. For microorganisms, tryptophan can act as a substrate for the production of cofactor for NAD [74]. Moreover, some tryptophan microbial derivates, e.g., tryptophol and indole lactic acid, exhibit the potential to shape microbiota by acting as quorum-sensing molecules [75] or via their antibacterial and antifungal properties [76,77,78].

Recent studies have revealed that circulating microbial tryptophan catabolites may affect the homeostasis of the human body. Despite the multidirectional function of compounds derived in the indole pathway, including increasing GLP-1 production [79] and stimulating intestinal motility, activation of the aryl hydrocarbon receptor (AhR) by these substances seems to be their most important role in the context of dermatological diseases [17,80]. This receptor is widely expressed, especially in the skin, intestines, and lungs. In the skin, the AhR is extensively expressed in fibroblasts, keratinocytes, Langerhans cells, melanocytes, sebocytes, mast cells, and lymphocytes [81]. It is known that some microbial tryptophan metabolites, especially indole-3-carbaldehyde (I3A), can stimulate the AhR on keratinocytes [82], Langerhans cells [83], melanocytes [84], and fibroblasts [85]. The effect of stimulation seems dose- and ligand-dependent. Different ligands trigger the interaction of the AhR with different transcriptional molecules, thereby inducing several biological effects [81].

Stimulation of the AhR in the skin can cause multidirectional reactions. In keratinocytes of atopic dermatitis patients, AhR activation was found to be related to the upregulation of filaggrin and loricrin, i.e., key proteins which build the skin barrier. Restoration of the skin barrier can be observed as a decrease in TEWL [80,86]. Moreover, activation of the AhR by tryptophan metabolites can improve wound healing and decrease scar formation by upregulating metalloproteinases and suppressing type I collagen and fibronectin expression, directly on dermal fibroblasts [87]. The stress-alleviating effect of the AhR pathway was also found in response to UV [88]. Indole pyruvate (IPyr), one of the bacterial metabolites of tryptophan, was found to exert a protective effect on keratinocytes exposed to UVB, in which the amounts of secreted IL-1b and IL-6 were similar to those of unirradiated cells [89]. Additionally, in a mouse model, an increase in TEWL and expression of IL-1b after irradiation was less marked in the group which had received IPyr topically [89]. Furthermore, indole-3-propionic acid (IPA), also known as the microbiota metabolite, can reduce oxidative stress in response to exogenous stressors [90]. It was found that the indole derivatives, indole lactic acid (ILA) and indole-3-carbaldehyde (I3A), might be involved in the induction of tolerance to allergens, especially food allergens. Due to the stimulation of the AhR, these compounds were able to reprogram intestinal CD4^+^ cells to known tolerogenic double-positive CD4^+^CD8αα^+^ cells [91].

Interestingly, the therapeutic effect of coal tar is related to the stimulation of AhR, which induces epidermal differentiation, neutralizes reactive oxygen species, restores filaggrin expression, and improves skin barrier proteins [92]. The importance of the AhR pathway in dermatology was emphasized by the discovery of the novel drug tapinarof, which acts as an AhR agonist [93]. Tapinarof has passed phase 3 of a clinical trial in psoriasis, and it has gained FDA approval for plaque psoriasis treatment [94,95]. Tapinarof is also undergoing several phase 3 clinical trials in adults and children with atopic dermatitis [95,96]. The promising effect of activation of the AhR suggests that tryptophan metabolites could exert similar therapeutic effects and could also be a useful tool in the management of dermatological diseases.

### 3.1. Atopic Dermatitis and Hypersensitivity Reactions

In the context of atopic diseases, there are a growing number of studies describing the function of tryptophan microbiota metabolites. Fang et al. revealed that administration of *Bifidobacterium longum* probiotics in atopic dermatitis patients can significantly decrease clinical symptoms measured in SCORAD and DLQI scales compared to a placebo. In the follow-up, the study group was characterized by lower IgE levels. Moreover, the responders were characterized by a significant increase in the serum levels of bacterial tryptophan metabolites, indole lactic acid (ILA), and indole-3-carbaldehyde (I3A) after the intervention. Additionally, fecal I3A was higher. It needs to be emphasized that a significant negative correlation was found between I3A level disease severity as measured with SCORAD and DLQI [97]. The following parts of the experiment confirmed the results in the mouse model and revealed that oral administration of I3A attenuated the activity of disease measured in the reduction in skin thickness, IgE levels, and concentrations of interleukins associated with the Th2 axis (TSLP, IL-4, IL-5) [97]. Another study, conducted by Yu et al., has reported corresponding results. Firstly, skin concentrations of I3A were significantly lower in patients with atopic dermatitis both in lesioned and non-lesioned skin [82]. The following part of the study confirmed the mentioned results in the mouse model. The topical and oral administration of I3A was effective in alleviating symptoms, e.g., skin thickness or itch, and induced a marked reduction in IL-4, IL-5, IL-6, IL-13, IL-22, and TSLP expression in skin cells. A decrease in TSLP expression after I3A treatment was also observed directly in keratinocytes. Interestingly, the effects of I3A administration included inhibition of inflammatory cell infiltration [82]. The decrease in TSLP expression found in both studies seems to be crucial in improving symptoms of atopic dermatitis. This cytokine is known as an orchestrating factor of the Th2 response and is responsible for the induction of atopy-related cytokines including IL-4, IL-5, IL-6, and IL-13 [98]. Another tryptophan metabolite, formylindolocarbazole (FICZ), can be synthesized from bacterial-derived indole-3-acetaldehyde (I3A) or indole-3-pyruvate (I3Pyr), especially under UV light [88,99]. FICZ can stimulate filaggrin expression and its abundance in human keratinocytes [86,100] and keratinocytes of a mite-induced AD-like NC/Nga murine model [100]. The mentioned model is an established model of AD [101,102]. Additionally, topical FICZ treatment reduced transepidermal water loss (TEWL) and significantly improved dermatitis [100]. IL-4-induced filaggrin downregulation in mRNA levels was reversed [100]. Skin epithelial barrier damage, manifested by increased TEWL and a decreased amount of filaggrin, is associated with AD pathogenesis. Allergens passing through damaged skin can stimulate immune cells and, in consequence, further damage the epithelial barrier, constituting a vicious circle of exposure [32].

Additionally, hypersensitivity reactions are associated with specific Trp metabolite alteration. Allergic subjects have decreased levels of indole-3-butyric and indole-3-lactic acids [103]. Additionally, new reports show that direct administration of some Trp metabolites can be useful in the management of delayed-type hypersensitivity [104]. Counterintuitively, FICZ in this study was related to the exacerbation of delayed-type hypersensitivity in a mouse model [104]. However, the evidence is not robust, and additional studies should be carried out to clarify the bacteria-derived indoles in allergy and their potential use in dermatology.

### 3.2. Lupus

By far, most reports of tryptophan microbiota metabolites have come from work on SLE. In SLE patients, an increased kynurenine-to-tryptophan ratio was observed [105,106,107], which exhibited a strong positive correlation with neopterin, a marker of immune activation [106]. Furthermore, the higher kynurenine-to-tryptophan ratio was associated with a worse working memory and poor visuospatial processing test results, severe fatigue, active disease, decreased complement, and anemia [105,106,107]. SLE was also linked with decreased indole-3-propionic acid [108]. The mentioned results suggest accelerated Trp metabolism in the kynurenine pathway at the expense of the indole pathway, which may originate from intestinal microbiota disturbances [109]. The mouse studies show similar shifts in Trp metabolism [109,110]. Correspondingly to humans, kynurenine seems to favor pro-inflammatory T-cell phenotypes in the lupus-prone mouse model [109]. Moreover, it was observed that a diet low in tryptophan was associated with slowing down the progression of the disease, which prevented the development of anti-dsDNA IgG, injury of the kidneys, increased Treg cells activity, and decreased mTOR kinase activation [109,110]. This suggests that interventions based on decreasing the dietary intake of tryptophan may be useful in the management of the disease.

## 4. Trimethylamine (TMA) and Trimethylamine N-oxide (TMAO)

Various compounds of this group are products of intestinal microflora degradation of ingested small molecules with a quaternary amine group, e.g., choline, L-carnitine, or phosphatidylcholine, which are abundant in eggs, liver, dairy products, and peanuts [15]. Trimethylamine N-oxide (TMAO) is the most recognizable and studied of all monoamines. TMAO is produced by the bacterial metabolite trimethylamine (TMA), which is oxidized by the liver. Mouse studies have reported that the intestinal microbiota is essential to TMA formation, and the production of TMA and TMAO can be almost completely suppressed by broad-spectrum antibiotics [111,112]. There is ample evidence that some genera of intestinal bacteria are associated with higher TMAO concentrations in plasma. For example, bacterial genera such as *Clostridia*, *Proteus*, *Shigella*, and *Aerobacter* are involved in the production of TMA, especially the strains *Anaerococcus hydrogenalis*, *Escherichia fergusonii*, *Clostridium hathewayi*, *Clostridium asparagiforme*, *Edwardsiella tarda*, *Proteus penneri*, *Clostridium sporogenes*, and *Providencia rettgeri*. Microbiota rich in the *Prevotella* genus, which is related to dysbiosis caused by a high-fat diet, is associated with higher plasma TMAO levels. What is more, the link between microbiota characterized by a higher level of the Bacteroides genus and lower levels of this metabolite in plasma has been proved [15,113].

Compared to previous substances, specific receptors for TMAO are still poorly known. TMAO is reported to bind to Protein Kinase RNA-Like ER Kinase (PERK) at physiological concentrations and to induce the FoxO1 transcription factor, which is the key factor in metabolic syndrome [18]. The harmful effect of this compound on the human organism is attributed to the acceleration of atherogenesis due to endothelial dysfunction, induction of oxidative stress, insulin resistance, and impairment of lipid metabolism [114]. There have been multiple studies describing its pro-inflammatory effect and a high TMAO level linked to persistent low-grade inflammation. The subjects characterized by high plasma TMAO levels have an elevated concentration of TNF, sTNF-R p75, and sTNF-R p55 [115]. Moreover, higher levels of TMAO in plasma are associated with overexpression of pro-inflammatory cytokines such as TNF, IL-6, and C-reactive protein, and decreased expression of anti-inflammatory cytokine IL-10 [15,116,117,118,119]. By induction of NLRP3 inflammasome activation, TMAO contributes to an increase in the levels of IL-1β and IL-18, which are crucial cytokines in various dermatological and rheumatological conditions [118,120]. Inhibition of the mentioned inflammasome supposedly decreases the detrimental effect of TMAO [121]. Because of NLRP3 inflammasome activation, lysosomal dysfunction and redox dysregulation of the cell occur, which results in the production of reactive oxygen species (ROS). Reactive oxygen species can damage the endothelium and disrupt the microenvironment of various organs, e.g., the intestine or the skin. Simultaneously, they can accelerate cellular senescence [120,122]. Additionally, it has been observed that TMAO can activate macrophages and monocytes, especially in the NLRP3 inflammasome pathway [118,121]. It is known that TMAO may enhance macrophage infiltration, and it may cause M1 polarization as well as Th1 and Th17 differentiation [121]. A higher TMAO concentration is associated with insulin resistance [123,124]. Furthermore, it is a factor of increased cardiovascular risk and accelerating atherosclerosis, and is also associated with a worse prognosis of the mentioned conditions [125,126].

### Dermatological Context

In dermatological conditions, the impact of TMAO still remains elusive. However, more and more studies are being published on this subject, and the results are encouraging. Sikora et al. found that patients suffering from psoriasis have significantly greater TMAO concentrations in plasma compared to control subjects. Moreover, increasing TMAO concentrations were associated with a greater abundance of conditions characteristic of metabolic syndrome such as hyperlipidemia, hypertension, obesity, non-alcoholic fatty liver disease, and a higher cardiovascular risk as measured with various scales, e.g., SCORE, Framingham Risk Score, or AHA/ACC. Furthermore, TMAO was an independent predictor of increased cardiovascular risk in patients with psoriasis, even after adjustment for classical risk factors [127]. Consistent results obtained in the study by Sun et al. confirm elevated TMAO levels in patients with psoriasis. Additionally, a significant correlation between TMAO and PASI scores and elevated concentrations of the TMAO precursor betaine in the psoriatic group have been found [128]. One of the most common complications of psoriasis is psoriatic arthritis (PsA). So far, there has been one study describing the role of circulating TMAO in PsA. According to the authors, there are significant positive correlations between TMAO levels and the severity of the disease, as measured by both skin and joint activity scores. Additionally, a positive correlation between TMAO levels and CRP levels has been found, which supports the proinflammatory role of TMAO [129].

TMAO concentration was increased in HS patients and correlated with disease severity (r = 0.57). This association was still evident after adjusting for common confounding covariates [130]. Both psoriasis and hidradenitis suppurativa are considered to increase cardiovascular risk and have similar comorbidities, including diabetes, hypertension, dyslipidemia, and inflammatory bowel diseases [131,132]. All the facts mentioned above suggest that an elevated TMAO level may not only affect the course of inflammatory skin diseases but can also modify their comorbidities.

TMAO impacts the pathogenesis of autoimmune- and immune-mediated diseases. Elevated TMAO levels affect patients with systemic lupus erythematosus (SLE), one of the most recognizable autoimmune diseases [133]. In a recent study, González-Correa et al. found that increased TMA and TMAO levels (5- and 8-fold, respectively) similarly occurred in a mouse model of SLE compared to the control group. Furthermore, they also discovered that a reduction in TMAO levels induced by the use of the trimethylamine lyase inhibitor 3,3-dimethyl-1-butanol (DMB), which inhibits bacterial synthesis of TMA, is related to a marked reduction in both TMA and TMAO and a less severe course of the disease in the animal model [134]. Decreased TMAO levels induced by DMB are associated with a reduction in proteinuria and inhibition of the development of hypertension induced by IMQ. It is also connected with the partial prevention of the increased plasma anti-dsDNA autoantibodies and IFNα mRNA levels, and normalization of T-cell imbalance. Additionally, strong positive correlations have been found between TMAO and systolic blood pressure and anti-dsDNA in plasma. DMB treatment has restored nrf2 levels and downstream antioxidant enzymes, suggesting *nfr2* downregulation is distinctive for higher TMAO levels and can contribute to oxidative stress in this fashion [134]. Despite these interesting results, the exact mechanism remains elusive.

To date, TMAO levels in systemic sclerosis (SSc) have not been studied yet. It is only known that elevated levels of the TMAO precursor, betaine, have been observed in this disorder [135]. However, recently, some engaging results have appeared from pre-clinical studies on the impact of TMAO on fibrosis in systemic sclerosis and skin physiology. TMAO can induce myofibroblast differentiation [136]. In isolated human fibroblasts, TMAO substantially increased cellular F-actin fibers and the expression of the genes responsible for the production of procollagen I and fibronectin [136]. Furthermore, skin fibroblasts exposed to TMAO were characterized by an increase in the secretion of TGF-β1, fibronectin, and procollagen I [136]. Additionally, the skin of SSc patients was characterized by 1.69- to 4.29-fold-higher FMO3 expression compared to control skin [137,138,139], which suggests that cell autonomous upregulation of FMO3 in SSc fibroblasts might contribute to their elevated collagen production underlying fibrosis.

In graft-versus-host disease (GVHD), in which pathogenesis resembles an autoimmune disease, TMAO can aggravate severity and mortality. This effect is caused by additional stimulation of autoreactive allogenic T cells by TMAO and can be seen in greater cytokine expression by these cells, especially IFN-γ, IL-17, and transcription factors STAT4 and STAT3 [121].

In spite of the promising results of the mentioned studies, more data are necessary to understand the role of bacterial metabolite TMAO and take clinical advantage of this knowledge.

The most important results of studies on metabolites in dermatological diseases are summarized in Table 1 at the end of the review.

## 5. Future Perspectives

The field of intestinal microbiota metabolites is rapidly developing. A comprehensive description of available data on lesser-known metabolites is beyond the scope of this review. However, it is worth mentioning bile salts, the group of secondary bile acids modified in the gut by microbiota. These compounds are recognized as anti-inflammatory factors, and their positive effects can potentially decrease the severity of psoriasis and atopic dermatitis [140,141,142,143].

**Figure 2:**
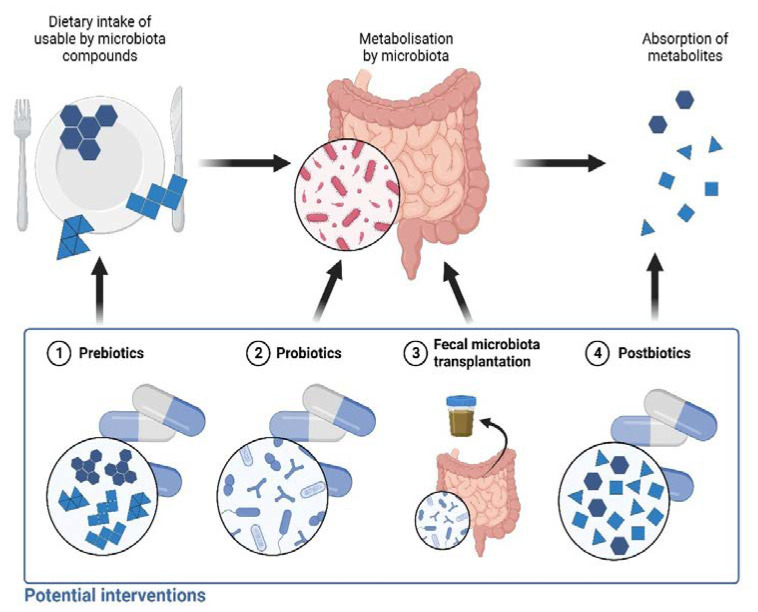
Production of microbiota metabolites and potential treatment options at various steps of this process. Ingested food is rich in compounds that can be utilized by intestinal microbiota. End products of microbiota metabolism are absorbed into the circulation. 1. Prebiotics, e.g., fructooligosaccharides and inulins, can be supplemented to achieve growth of beneficial microorganisms and an increase in production of eligible metabolites. 2. Probiotics. 3. Fecal microbiota transplantation is carried out with the aim to replace pathogenic bacteria with health-promoting ones. 4. Postbiotics consist of favorable metabolites of intestinal microbiota. Created with BioRender.com.

The mentioned studies emphasize the role of the microbiome and its metabolites in the pathogenesis of various conditions. Consequently, modifying microbiota seems to be a promising therapeutical option (Figure 2).

Treatment alternatives may include direct modifications of microbiota, indirect modifications, or only supplementation of beneficial metabolites (Figure 2). Direct modifications include the administration of probiotics or fecal microbiota transplantation (FMT). Indirect modification can be focused on the administration of dietary elements, so-called prebiotics, which can be metabolized by bacteria for nutritional purposes. Prebiotics can favor some beneficial bacterial strains and be precursors of health-promoting bacterial metabolites. One novel approach involves the direct use of beneficial bacterial metabolites, especially SCFAs. Health supplements composed of beneficial metabolites are termed postbiotics. The mentioned methods in the context of dermatological conditions are briefly discussed below.

### 5.1. Prebiotics

Prebiotics are dietary ingredients that support the growth or activity of beneficial bacteria. This group consists of various dietary-derived substances such as galactooligosaccharides, fructooligosaccharides, fiber, beta-glucans, pectins, gums, resistant starch, etc. [144]. Supplementation of such substances can potentially be useful in the management of dysbiosis [145]. In addition, human milk oligosaccharides support infants’ development of a balanced gut microbiota composition; this is interpreted by some as a risk factor for allergies [146]. The beneficial impact of prebiotics can be a result of an increase in the production of health-promoting bacterial metabolites, i.e., SCFAs [147].

Benefits from the supplementation of prebiotics are most marked in atopic dermatitis. Prebiotic treatment significantly decreased the incidence of AD in patients, according to a meta-analysis of 22 clinical trials using prebiotics in newborns to avoid allergies [148]. Although some trials have indicated encouraging outcomes, there is little information available about the effectiveness of prebiotics in the treatment of AD. However, two modestly sized randomized controlled trials reported an improvement in symptoms of the disease [149,150].

In the context of allergy, there is some evidence of the beneficial role of prebiotics in the prevention of such diseases [151]. In line with this, the World Allergy Organization advises prebiotic supplementation in newborns who are not exclusively breastfed, emphasizing that the quality of the evidence is poor [152].

For autoimmune diseases, the evidence is even more scarce. However, studies in psoriasis and systemic lupus erythematosus found that supplementation of synbiotics containing fructooligosaccharides can alleviate disease symptoms and improve inflammatory status [153,154].

Despite the limited data, prebiotics have an excellent safety profile with no notable adverse effects reported in the revised literature.

### 5.2. Probiotics

Probiotics are living microorganisms that, when given in sufficient quantities, have a positive impact on the host organism [155]. The effects of probiotics seem to be strain- and dose-dependent, and with a multitude of bacterial genera, these aspects make research particularly hard to interpret [156]. Despite this knowledge, recommendations published by the most recognized scientific societies often consider the effect of probiotics as a group, neglecting their wide heterogeneity. However, recent studies report that this group potentially can be applied in many fields of dermatology.

Several large-cohort randomized controlled trials have investigated the value of oral probiotics for the treatment and prevention of AD. A recent meta-analysis of eleven randomized controlled trials including 2572 infants found that intake of *Lactobacillus rhamnosus* during pregnancy and thereafter significantly lowered the risk of developing AD when assessed at 2 years out and 6–7 years out (RR 0.60 and RR 0.62, respectively) [157]. What is more, the results from other meta-analyses support the use of oral probiotics for the treatment of AD both in children and adults [158,159,160]. The mixture of strains *Bifidobacterium animalis* subsp *lactis* CECT 8145, *Bifidobacterium longum* CECT 7347, and *Lactobacillus casei* CECT 9104 was associated with the best results in the pediatric population, and the mixture of *Lactobacillus salivarius* LS01 and *Bifidobacterium breve* BR03 has the best results in adults [158,159,160]. Similar to AD, some probiotics may be effective in the prevention and treatment of allergies [161,162]. 

The beneficial effects of probiotics in autoimmune- and immune-mediated diseases appear to be connected to a decline in pro-inflammatory indicators, such as the production of IL-6 and CRP or the activation of Th1 and Th17 cells [163]. Additionally, recent studies imply that an anti-inflammatory effect of probiotics may result from their ability to improve gut permeability [164]. In contrast to findings in allergy and atopic dermatitis, the impact of this group on the prevention of autoimmunity is still poorly understood, despite the recent research suggesting that probiotics may also be helpful in this indication. Administration of some bacterial strains has been associated with protection against induced encephalitis in mouse models [165]. In the context of the treatment, available human research indicates that certain bacterial strains may be helpful in several dermatological disorders, such as psoriasis, SLE, and SSc.

Several randomized controlled studies have investigated the effectiveness of oral probiotics for the treatment of psoriasis. In one of them, administration of a multi-strain mixture including *Lactobacillus acidophilus*, *Bifidobacterium bifidum*, *Bifidobacterium lactis*, and *Bifidobacterium longum* with 1.8 × 10^9^ colony-forming units (CFUs) led to a significant decrease in PASI, Psoriasis Symptom Scale, and DLQI scores compared to the placebo group (MD −5.55, −4.84 and −9.87, respectively) [166]. Additionally, the intervention was linked to a notable reduction in inflammatory markers, including hs-CRP and IL-6, as well as an increase in total antioxidant capacity [166]. The use of *Streptococcus salivarius* K-12 was also associated with a significant reduction in psoriasis symptoms [167]. The PASI 75, PASI 90, and PASI 100 response was achieved in 84%, 75%, and 55% of patients in the study group, respectively, compared to 42.8%, 30%, and 12.4% in the control group [167].

Apart from the improvement in PASI, a multi-strain preparation contributed to a decrease in inflammatory markers and an enhanced gut barrier, which was measured as lipopolysaccharide (LPS) translocation [168].

Probiotics have also been shown to have a beneficial impact on SLE in a recent randomized, double-blind, placebo-controlled trial [154]. The intervention included the administration of a preparation consisting of fructooligosaccharides and a mixture of *Lactobacillus* and *Bifidobacterium* strains. In the study group, after 60 days of supplementation of the preparation, significantly lower hs-CRP levels and a decrease in the IL-6 and SLE disease activity index 2K was noticed [154]. Interestingly, significant increases in butanoate or butyrate metabolism were seen in the post-synbiotic group and pre-synbiotic group compared to the pre-intervention-administered preparation group and the placebo group, according to functional prediction based on gut microbiota profiling [154].

Probiotics are not only beneficial in decreasing the severity of a disease, but they can also alleviate gastrointestinal symptoms. Despite having no effect on disease activity, probiotics were linked to an improvement in reflux symptoms in SSc patients [169].

### 5.3. Fecal Microbiota Transplantation

FMT is the most direct way of restoring the balance of the gut microbiota. Most data in dermatology come from FMT in atopic dermatitis. AD therapy with FMT has shown promising results. Zhou et al. reported a case of complete remission of AD in a 31-year-old female patient with concomitant functional constipation after FMT. It has also been noticed that the clinical improvement was accompanied by changes in the gut microbiota, especially a decrease in the abundance of bacteria involved in metabolic disorders and those that promote inflammation [170]. Due to the case report character of this study, the conclusions on the efficacy of FMT for treating AD are limited. A cross-over pilot study that included nine participants with moderate or severe AD showed a marked reduction in symptoms as measured by SCORAD score. The average SCORAD score decreased by approximately 85%. A 50% and 75% decrease in the SCORAD score was obtained by seven and six patients, respectively [171]. Furthermore, during the follow-up period, the frequency of weekly topical corticosteroid use decreased by 90% [171]. It was also observed that the higher the similarity between the microbiome of donors and the patients after FMT, the better the clinical improvement [171]. A study carried out by Zou et al. showed the long-term safety of FMT in AD in children. In a group of six children with AD, they observed the peak SCORAD score decrease after 3 months after FMT, followed by a gradual increase after the 6th month [172]. Three clinical remissions (defined as SCORAD ≤ 5 or a decrease of more than 30 points) and two clinical improvements (defined as a decrease in the SCORAD index of more than 10 points) were observed then. What is notable about the mentioned studies is the fact that no adverse events were noted and FMT was considered a safe procedure.

Promising results for the use of FMT in SLE come from a study by Huang et al. FMT was performed in 19 patients with SLE failing to respond to usual therapy for 8 weeks. After the intervention, there was observed a significant reduction in the mean SLEDAI-2K score from 9.45 ± 3.97 to 6.61 ± 4.43 at baseline. At the primary endpoint of week 12 after FMT, SRI-4 response was reached by 42.12% of patients, and a significant decrease in the level of serum anti-dsDNA antibodies was observed [173]. Furthermore, the microbiota of the patients increased in diversity, especially in SCFA-producing genera, and fecal SCFAs concentrations rose [173]. These results suggest that an induced increase in exposure to SCFAs can be at least partially related to clinical improvement.

In contrast, in research on the impact of FMT on SSc, no significant difference in SCFA concentrations after FMT was noticed [174]. However, FMT was found to relieve gastrointestinal symptoms, which are common complications of SSc [174].

Despite primary success in the case of a patient successfully treated with FMT [175], less encouraging results have been provided by Kragsnaes et al. in their study on FMT in psoriatic arthritis [176]. The study group consisted of 15 and 16 volunteers in FMT and placebo groups, respectively. FMT was found to slightly increase treatment failure, defined as a worsening of symptoms that required enhancement of the treatment [176].

Data on other immune-mediated skin conditions are limited. For psoriasis, there is a case report of a 36-year-old man who experienced an improvement in received the body surface area (BSA), PASI, dermatology life quality index (DLQI), intestinal symptoms, and serum level of TNF after FMT [177].

What is notable about the studies described above is that the FMT procedure was considered safe and the noticed adverse events were mild and transient, i.e., diarrhea, bloating, and abdominal pain 170–173,176]. Two major adverse effects in the form of duodenal perforation and laryngeal spasm were observed only in the SSc study [174]. Of note, adverse events were significantly method-dependent and endoscopic microbiota transplantation was associated with greater complications than transplantation with the use of capsulated microbial concentration [170,171,172,173,176].

It is worth noticing that there is still little information available on donor matching, which appears to be the most important factor in the context of the therapeutic success of FMT. The donor is chosen intuitively by the exclusion of potentially contagious infections rather than based on specific tests, which could potentially indicate the most adequate combination of donor and recipient. Additionally, there are currently no relevant standards for screening donor microbiota. The more we know about factors influencing the response to FMT, the better clinical results can be achieved.

### 5.4. Postbiotics

It is still barely known how the administration of bacterial metabolites affects human health. However, there is a whole range of supplements available on the market, especially those based on sodium butyrate. Even now, there are no data on the use of such supplements in dermatology. Available human studies prove that oral administration of SCFAs is safe and can be beneficial for health. In metabolic syndrome, after 4 weeks of oral administration of 4 g of butyrate, a dampened response of macrophages to oxLDL, which manifested as decreased TNF and IL-6 production, was observed [178].

Encouraging results come from randomized, placebo-controlled studies on type 2 diabetes, which is a common complication of certain dermatological diseases, e.g., psoriasis and psoriatic arthritis [179,180]. Butyrate taken in 600 mg daily doses significantly decreased oxidative stress by increasing the total antioxidant capacity (TAC) and activity of superoxide dismutase (SOD) of the blood [181]. Furthermore, butyrate exerted an anti-inflammatory effect by reducing the amount of hs-CRP, TNF expression, fasting blood glucose, and inflammasome-associated proteins, which suggests that butyrate can suppress low-grade inflammation associated with diabetes [182,183]. Similarly to butyrate, propionate supplementation was found to decrease fasting glucose and improve insulin sensitivity and the lipid profile in obese adults [184,185,186]. Additionally, propionate administration was related to an increase in resting energy expenditure and lipid oxidation in fasted humans [187].

Interestingly, in the case of Trp metabolites, there are several human studies available, especially on the effects of supplementation of indole-3-carbinol (I3C). I3C is considered a dietary product rather than a bacterial metabolite. However, it is known that indole-3-carbinol can be converted into indole-3-aldehyde and vice versa [188,189]. It has been found that supplementation of I3C is an effective and valuable adjuvant treatment, especially in HPV-associated infections, i.e., recurrent respiratory papillomatosis and vulvar intraepithelial neoplasia [190,191]. The administration of I3C’s precursor, 3,3-Diindolylmethane, has been found to induce regression of cervical intraepithelial neoplasia [192]. Although these results are promising, no human studies have been conducted on the effects of other indole metabolites.

**Table 1:** A review of studies available on the subject of the impact of intestinal microbiota metabolites on dermatological conditions.

References	Disease	Studied Model	Main Findings
Short-chain fatty acids (SCFAs)			
Schwarz et al. [61]	Psoriasis	imiquimod-induced psoriasis-like skin inflammation mouse model, skin biopsies from patients with psoriasis	Topical administration of sodium butyrate (SB) reduced symptoms of psoriasis-like skin inflammation Topical SB increased the number and activity of Treg cells, IL-10 transcription, and decreased IL-17 transcription in the skin of a mouse model SB increased the transcription of IL-10 and decreased the transcription of IL-6 and IL-17 in human skin biopsies
Krejner et al. [62]	Psoriasis	skin biopsies from patients with psoriasis	Ex vivo treatment with SB caused a reduction in IL-17 and IL-6, and an upregulation of IL-10 transcription in skin biopsies Skin with psoriasis has decreased expression of GPR109a and GPR43, SB upregulates these receptors
Rodríguez-Carrio et al. [67]	Systemic lupus erythematosus	21 SLE patients, 25 healthy individuals	Fecal acetate and propionate are higher in patients with SLE compared to controls Dysbiosis in SLE patients
Sanchez et al. [28]	Systemic lupus erythematosus	MRL/lpr and NZB/W F1 lupus-prone mice	Oral mixture of sodium butyrate and sodium propionate reduced local and systemic antibody responses Orally administered SCFAsT reduced lupus skin lesions and kidney pathology
He et al. [68]	Systemic lupus erythematosus	MRL/lpr lupus-prone mice	Reduction in microbial diversity in the SLE mouse model Oral administration of SB reduced renal histopathological changes and increased microbiota diversity
Patrone et al. [69]	Systemic sclerosis	18 SSc patients, 9 healthy subjects	Dysbiosis manifested as a decrease in butyrate-producing genera more prominent in patients with gastrointestinal involvement
Park et al. [71]	Systemic sclerosis	bleomycin-induced fibrosis mouse model of SSc, human dermal fibroblasts	SB administered orally or subcutaneously reduced bleomycin-induced dermal and lung fibrosis SB treatment inhibits TGF-β1-induced fibrotic responses in human dermal fibroblasts
Reddel et al. [35]	Atopic dermatitis	19 children with AD and 18 healthy individuals	AD was characterized by dysbiosis, especially manifested in the depletion of butyrate-producing bacteria
Nylund et al. [36]	Atopic dermatitis	28 infants with atopic dermatitis and 11 healthy infants	Less severe eczema was associated with increased butyrate-producing bacterial abundance and microbiome diversity
Song et al. [39]	Atopic dermatitis	90 patients with AD and 42 volunteers without AD	Decreased fecal level of butyrate and propionate in AD patients Some subspecies of *Faecalibacterium prausnitzii* are linked with AD
Lee et al. [38]	Atopic dermatitis	234 patients with mild to severe AD, 112 non-AD subjects	Diversity of the microbiota in moderate to severe AD was significantly lower than in non-AD Disordered gut microbiota development in AD was associated with dysregulated SCFA production
Roduit et al. [40]	Atopic dermatitis	301 one-year-old children	Children with the highest fecal levels of butyrate and propionate were less prone to atopic sensitization and were less likely to develop asthma between the ages of 3 and 6 Food allergies and allergic rhinitis were less common in children with the highest butyrate levels
Cheng et al. [42]	Atopic dermatitis	75 infants	Low fecal butyric acid was associated with an increased risk of developing atopic dermatitis, food sensitization, and wheezing up to 8 years old
Gio-Batta et al. [44]	Atopic dermatitis	65 infants	A lower level of valeric acid at 3 years of age was associated with a higher prevalence of atopic eczema at the age of 8 years
Gio-Batta et al. [45]	Atopic dermatitis	110 one-year-old children	Eczema at 13 years of age was inversely correlated with the amount of fecal valeric acid at 1 year of age
Folkerts et al. [52]	Allergy	Human mast cells	Propionate and butyrate inhibited IgE- and non-IgE-dependent human mast cell degranulation
Schwarz et al. [57]	Contact dermatitis	sensitized C57BL/6J mice	Sodium butyrate (SB) administered topically or subcutaneously inhibited both the elicitation phase and ongoing contact hypersensitivity response SB induced the anti-inflammatory response via an increase in the number of skin Treg cells and an increase in IL-10 transcription
Trompette et al. [33]	Atopic dermatitis	atopic dermatitis-like skin inflammation mouse model	Fermentable fiber-rich diet or orally administered sodium butyrate alleviate systemic allergen sensitization and disease severity Oral butyrate stimulates terminal differentiation of epidermal keratinocytes and promotes skin barrier function
Tryptophan metabolites			
Tsuji et al. [86]	Atopic dermatitis	normal human epidermal keratinocytes	The activation of AHR by tryptophane metabolite significantly increased filaggrin expression FICZ reversed the IL-4-induced downregulation in transcription and protein levels of filaggrin
Aoki et al. [89]	UVB-induced skin damage	HR-1 mice, HaCaT keratinocytes	Topical application of indole-3-pyruvate reduced the severity of UVB-induced skin lesions, the augmentation of dermal thickness, and transepithelial water loss Suppression of the overproduction of IL-1b and IL-6 in response to UVB radiation in a mouse model Indole-3-pyruvate improved the survival rate and reduced the expression of IL-1b and IL-6 in UVB-exposed HaCaT keratinocytes
Fang et al. [97]	Atopic dermatitis	87 patients with atopic dermatitis, sensitized female C57BL/6 mice	*Bifidobacterium longum* probiotic treatment increased serum and fecal indole-3-carbaldehyde, significantly reduced AD symptoms Indole-3-carbaldehyde displayed a significant negative correlation with atopic dermatitis severity measured in both SCORAD and DLQI Oral administration of indole-3-carbaldehyde alleviated AD-like skin lesions in sensitized mice
Yu et al. [81]	Atopic dermatitis	19 patients with AD, 19 healthy volunteers, sensitized C57BL/6 and BALB/c mice	Decreased indole-3-aldehyde was observed in both lesional and non-lesional skin of AD patients Topical and orally administered indole-3-aldehyde attenuated MC903-induced AD-like dermatitis in mouse and decreased expression of IL-4, IL-5, IL-6, IL-13, and TSLP Topically administered indole-3-aldehyde reduced inflammatory cell infiltration in mice
Kiyomatsu-Oda et al. [100]	Atopic dermatitis	NC/Nga mice, HaCaT cells and normal human epidermal keratinocytes (NHEKs)	Tryptophan metabolite FICZ improved symptoms of AD-like dermatitis, decreased TEWL, restored filaggrin expression, reduced the number of infiltrated mast cells, and reduced expression of IL-22 and IFN-γ genes in a mouse model FICZ upregulated expression and abundance of filaggrin in HaCaT and NHEKs cells
Singh et al. [104]	Delayed-type hypersensitivity	Sensitized C57BL/6 mice	Topical administration of indole-3-carbinol and 3,3′-diindolylmethane alleviated symptoms, triggered induction of Tregs, and suppressed Th17 cells of delayed-type hypersensitivity in a mouse model FICZ exacerbated disease in a mouse model and suppressed Treg cells
Shinde et al. [108]	Systemic lupus erythematosus	48 patients with active SLE, 24 patients with SLE in remission, and 20 control subjects	Serum indole-3-propionic acid was significantly higher than in the control group
Trimethylamine N-oxide (TMAO)			
Sikora et al. [127]	Psoriasis	72 patients with psoriasis and 40 matched controls	In patients with psoriasis, serum TMAO was significantly higher than in the control group TMAO was found to be an independent predictor of cardiovascular risk
Sun et al. [128]	Psoriasis	180 patients with psoriasis, 60 healthy controls	Psoriatic patients had significantly higher serum levels of TMAO compared to controls TMAO had a positive correlation with PASI score
Coras et al. [129]	Psoriatic arthritis	38 patients with psoriatic arthritis	Serum TMAO demonstrated a significant correlation with indicators of disease activity for the skin and peripheral joints
Barea et al. [130]	Hidradenitis suppurativa	35 patients with hidradenitis suppurativa and 35 matched controls	Patients had increased serum TMAO levels compared to controls The level of circulating TMAO correlated positively with the HS Sartorius score also after adjustment for confounding factors Serum TMAO levels and PhA were the two primary indicators of the clinical severity of HS based on a linear regression model
Li et al. [133]	Systemic lupus erythematosus	17 patients with SLE and 17 healthy controls	Serum levels of trimethylamine N-oxide (TMAO) were found to be elevated in lupus patients compared to controls
González-Correa et al. [134]	Systemic lupus erythematosus	Imiquimod-induced mouse model of SLE	Plasma TMAO concentrations were significantly elevated in the serum of active systemic lupus erythematosus patients
Wu et al. [121]	Graft-versus-host disease	C57BL/6 and BALB/c mice	Induced by oral administration elevation of plasma TMAO was associated with worse course and survival of graft-versus-host disease

## 6. Conclusions

Taking into consideration that various microbiota metabolites have opposite functional properties, an approach based on multi-metabolite analysis and correlation with metagenomic analysis of the gut microbiota appears to be a particularly interesting direction for future research.

Intestinal microbiota metabolites have a significant impact on the pathogenic processes in dermatological diseases. SCFAs and indole-derived metabolites seem to act via their immunomodulatory and anti-inflammatory properties. Amine derivatives, particularly TMAO, can accelerate the progression of diseases and contribute to the development of complications because of their pro-inflammatory activity. Thus, modifications of microbiota, which may alter the metabolite concentrations, are a promising therapeutic option for several inflammatory dermatological diseases, potentially exhibiting a very good safety profile.

## Data Availability

Not applicable.
